# An integrated wet-spinning system for continuous fabrication of high-strength nanocellulose long filaments

**DOI:** 10.1038/s41598-023-40462-5

**Published:** 2023-08-12

**Authors:** Pooja S. Panicker, Hyun Chan Kim, Jaehwan Kim

**Affiliations:** 1 Creative Research Center for Nanocellulose Future Composites, Inha University, 100 Inha-ro, Michuhol-ku, Incheon, 22212 Republic of Korea; 2 University of Michigan, Ann Arbor, MI 48109 USA

**Keywords:** Mechanical engineering, Materials science

## Abstract

The continuous production of high-strength nanocellulose long filaments (NCLFs) is critical in natural fiber-reinforced polymer composites. Despite the widespread availability of numerous filament production processes, the cost-effective and continuous fabrication of high-strength NCLFs on a large scale remains an ongoing challenge. Herein, we present an integrated wet-spinning system by incorporating a few previously researched filament production techniques to mass fabricate high-strength continuous NCLFs. The spinning speed is increased to improve NCLF productivity, and the bobbin winder speeds, collector bobbin winder location, and NCLF drying conditions are tuned. At the spinning speed of 510 cm/min, a production rate of 4.99 m/min is achieved, five times higher than the productivity of the former pilot system (0.92 m/min). Moreover, an AC electric field and mechanical stretching are introduced to highlight the versatility of the proposed integrated wet-spinning system, thereby enhancing the mechanical properties of NCLFs.

## Introduction

Cellulose has been utilized in the form of fiber or its derivatives for more than a century. The advancement of nanotechnology has accelerated the extraction of cellulose fibers at the nanoscale, revolutionizing the field of cellulose research. Nanosized cellulose, called nanocellulose, has proven to be a high-performance building block of nature^1^. Nanocellulose exists in different forms depending on their geometric characteristics, such as length and diameter. Examples of these forms include cellulose microfibers (CMFs), cellulose nanofibers (CNFs), cellulose nanocrystals (CNCs), and cellulose nanoparticles (CNPs)^1,2^. CNFs possess unique characteristics such as biodegradability, biocompatibility, flexibility, lightweight, and a high aspect ratio, rendering them suitable for a wide range of applications such as energy storage, medicine, food packaging, cosmetics, structural composites, and healthcare^1,3^. The two primary strategies considered for preparing CNFs are top-down and bottom-up. The top-down strategy emphasizes the isolation of CNFs, CNCs, and CNPs from natural sources by utilizing several chemical and mechanical methods^4^. Although the isolation of CNFs is quite simple, its size is too small, limiting its applications for fibers and composites. Thus, extending it to a large-scale continuous filament, the so-called nanocellulose long filament (NCLF), is challenging.

The bottom-up approach focuses on the fabrication processes of NCLF, which include a wide range of spinning techniques. Solvent-spinning and melt-spinning are the most prevalent methods for producing synthetic and cellulose-based filaments. Wet-spinning, dry-spinning, and dry-jet wet-spinning are a few different solvent-spinning techniques^5,6^. Electrospinning is a widely reported method whereby fiber fabrication occurs under an electric field^7^. All spinning procedures commence with dissolving the polymer precursor to obtain a spinning dope (suspension) which is then extruded through a spinneret (nozzle). The wet-spinning process begins with extruding suspension through a nozzle of the desired diameter into a coagulation or precipitation bath to form filaments^8^. In dry-spinning, the solvent is evaporated using hot air following extrusion from the nozzle, whereas, in melt-spinning, the filaments are prepared by extrusion of the suspension followed by cooling^9^. Apart from these distinct spinning techniques employed for the fabrication of filaments, factors such as process parameters, chemical modification/treatments, mechanical stretching or twisting, and electric or magnetic field alignment can also be used to tune the properties of the resultant filament^10–12^. Wet-spinning by syringe extrusion is the most used spinning technique in this field of research since it provides flexibility in modifying the fabricated filaments’ structural, mechanical, and thermal properties. The coagulation for spinning filaments often includes electrolyte solution (NaCl, HCl, H_2_SO_4_, C_6_H_8_O_7_) or organic solvents such as acetone and ethanol^13,14^.

In recent years, wet-spinning techniques such as syringe extrusion and flow-focusing have become the most popular methods for spinning filaments due to their versatility and reliability, as they can be customized based on the required specifications, such as nozzle diameter, coagulant, size, and shape of coagulation bath, number of bobbin winders, and number of heating sources. In 2011, Walther et al. introduced a conventional wet-spinning process to fabricate nanofibrillated cellulose (NFC) microfibers by extruding NFC hydrogel into the coagulation bath containing ethanol, dioxane and isopropanol^8^. In 2017, Mohammadi et al. proposed a capillary extrusion spinning scheme to fabricate NFC by extruding the aqueous dispersion of CNF into a coagulation bath^15^. On the other hand, an intricate wet-spinning setup was developed by Kang et al. consisting of one coagulation bath, three washing baths, and thirteen rollers, including six elongation rollers, one heating roller, and one winder roller for the fabrication of carbon nanotube/polyvinylidene fluoride (PVDF) fibers^16^. Håkansson et al. achieved CNF alignment in a flow-focusing system utilizing a surface-charge-controlled gel transition in conjunction with hydrodynamics, while Nechyporchuk et al. and Wise et al. reported extrusion of CNCs and CNFs in a flow-focusing channel^17–19^. In addition, our research group recently introduced a custom-designed wet-spinning (pilot) system consisting of three bobbin winders, a coagulation bath, and a washing bath to continuously produce CNF-graphene oxide (CNGO) hybrid filaments. The system shows great potential and has gained recognition since it offers continuous production of CNF by adopting a facile wet-spinning process^20^. Furthermore, the influence of the alternating current (AC) electric field on CNF alignment was investigated by adopting the same wet-spinning setup with a few modifications ^21^. However, low filament output was a drawback of this pilot wet-spinning system, as it can only offer a maximum spinning speed (linear filament velocity as it exits the nozzle) of 100 cm/min. Thus, meeting the current NCLF productivity standards while maintaining their mechanical properties is a recurring concern.

For the first time, this study proposes a comprehensive approach for achieving continuous production of high-strength NCLFs with a superior production rate by employing an integrated wet-spinning system with ten bobbin winders, one coagulation bath, and one washing bath. The system also offers additional scope for incorporating electric field alignment, mechanical stretching, and surface coating of NCLFs, which demonstrates the versatility of the designed wet-spinning system. Spinning speed, bobbin winder speed, NCLF drying, and the position of the collector bobbin winder were discreetly optimized to achieve high-strength continuous NCLFs. Highly refined CNF suspension, referred to as fine CNF, was utilized to optimize the system, and citric acid, a weak organic acid, was selected as the coagulant due to its favorable low toxicity profile^22^. The study investigated several spinning speeds, such as 180, 250, 320, and 510 cm/min, to achieve varied NCLF production rates. The fabricated NCLFs were analyzed in terms of morphology and mechanical properties to evaluate the consistency and sustainability of the proposed wet-spinning system. Furthermore, the design of the integrated wet spinning system ensures its flexibility to produce filaments using various spinning techniques. It can also accommodate suspensions containing different materials, broadening the filament fabrication possibilities.

## Experimental procedure

### Materials

The 2 wt% TEMPO-oxidized CNF suspension (1 mmol/gm) was purchased from Moorim company, South Korea. The citric acid (99.9%) was purchased from Sigma-Aldrich Co. An inbuild laboratory purification system (D7429, Thermo Scientific) was used to collect Deionized (DI) water.36.

### Preparation of CNF suspension

The 2 wt% TEMPO-oxidized CNF suspension was diluted to 0.5 wt% and fractionated in an ultracentrifuge (CP80NX, Hitachi, Japan) for 3 h at 45,000 revolutions per minute (RPM) to obtain fine CNF suspension. The homogeneous middle layer obtained after fractionation is referred to as Fine CNF. This process was repeated under the same condition (45,000 RPM for 3 h) until a final concentration of 2 wt% Fine CNF suspension was achieved. The homogenous fine CNF suspension exhibits an average fiber width of 2 nm and an average fiber length of 640 nm^23^. Further, the zeta potential of fine CNF is analyzed (Figure S1) to investigate the uniform particle distribution of the fine CNF obtained. The 2 wt% fine CNF suspension was further homogenized in an ultra-turrax homogenizer (T-25, IKA, USA) for 10 min and transferred to a 50 mL clear syringe (Musashi Engineering, Japan). It was centrifuged in a high-speed centrifuge (Supra 22 K, Hanil Science Co., Ltd., South Korea) at 5,000 RPM for 30 min to eliminate air bubbles.

### The integrated wet-spinning system

**Figure 1:**
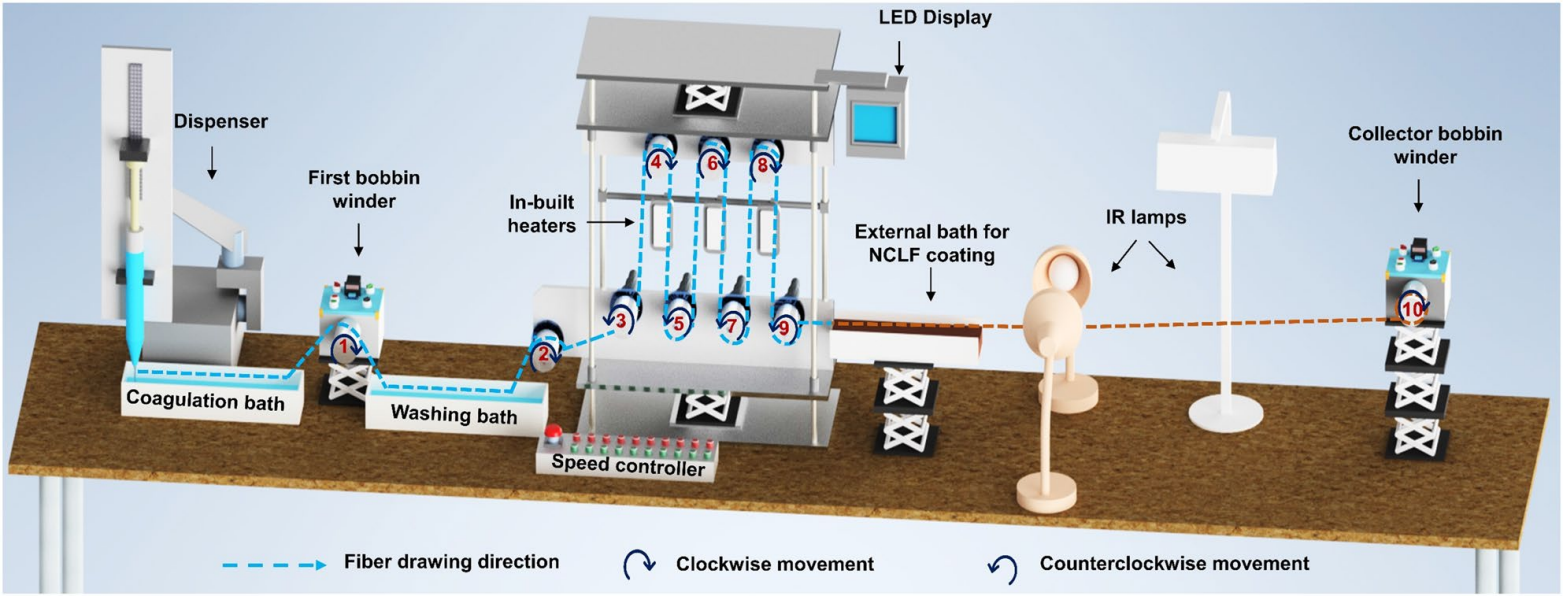
The integrated wet-spinning system for the fabrication of NCLF.

Figure 1 illustrates the integrated wet-spinning system designed to fabricate high-strength, continuous NCLF while attaining the targeted NCLF productivity. The integrated wet-spinning system comprises a CNF suspension dispenser, one coagulation bath, one washing bath, ten bobbin winders, three in-build heaters (placed between bobbin winders 3 to 9) with temperature sensors, three 250 W external infrared (IR) lamps (Philips, BR125, South Korea) (placed between bobbin winders 9 and 10), an LED display, and a speed controller.

The wet-spinning of CNF suspension was carried out using a precision fluid dispenser (SMP3-C, MUSASHI Engineering, Japan). A PTFE nozzle with an inner diameter of 250 µm and a length of 20 mm was utilized to extrude CNF suspension into the coagulation bath containing 0.2 wt% of the citric acid. Simple ion-exchange phenomena influence the coagulation of fine CNF suspension in the 0.2 wt% citric acid solution bath during the wet-spinning process to produce filaments^21^. The first bobbin winder carries the filaments formed in the coagulation bath to a washing bath containing DI water. The position and movement of the bobbin winders are carefully calibrated to execute the successful fabrication of continuous NCLFs. The second bobbin winder is positioned 65 cm from the first bobbin winder to accommodate the washing bath. The bobbin winders 3 to 9 are spaced 13–14 cm apart to allow the operator to handle filaments during NCLF fabrication. For the continuous movement of NCLFs from the first to the collector bobbin winder, bobbin winders 1, 2, 4, 6, 8, and 10 are programmed to rotate clockwise while bobbin winders 3, 5, 7, and 9 rotate counterclockwise. The collector bobbin winder is placed 2.35 m away from the ninth bobbin winder to adjust the drying time and shape of the resultant NCLFs. Since the position of the collector bobbin winder also plays a vital role in the morphology and mechanical properties of the fabricated NCLFs, the ideal position of the collector bobbin winder was experimentally optimized and kept at an acute angle from the ninth bobbin winder^24^. The speed controller comprises one emergency push button and ten individual red and green switches to increase and decrease the RPM of bobbin winders, respectively. The LED display shows the system’s ON/OFF controls, the RPM of each bobbin winder, and the temperatures of the three built-in heaters. It also provides options to SAVE/RECALL programs. Hence, after optimizing the speeds of bobbin winders to match the selected spinning speed, the RPMs of all ten bobbin winders, including the temperatures of three in-build heaters, can be saved as a program. The desired program can be recalled as per the operator’s need. Mechanical stretching of NCLFs can also be achieved by discrete control of the speeds of the preferred bobbin winders. Thus, the integrated wet-spinning system presents versatility in the processing, production, surface modification, and alignment of NCLFs.

The system also contributes flexibility to perform surface coating experiments on NCLFs by installing an external bath adjacent to the ninth bobbin winder. The operator can install or uninstall this external bath as desired. The integrated system can also accommodate an external amplifier (TERK 609A-1, PolyK Technologies, USA) and an arbitrary signal generator (Keysight 33220A, Agilent, USA) to facilitate the delivery of AC electric field in conjunction with the wet-spinning process for better alignment of NCLFs^21^. The surface coating of NCLFs by esterified poly(vinyl alcohol) − citric acid − lignin (E-PCL) resin using the integrated wet-spinning system has already been demonstrated by our research group^25^. To preserve the reproducibility of NCLFs, the integrated wet-spinning system is installed in a cleanroom under a constant temperature (25 °C) and relative humidity (40%).

### NCLF fabrication with parameter changes

**Figure 2:**
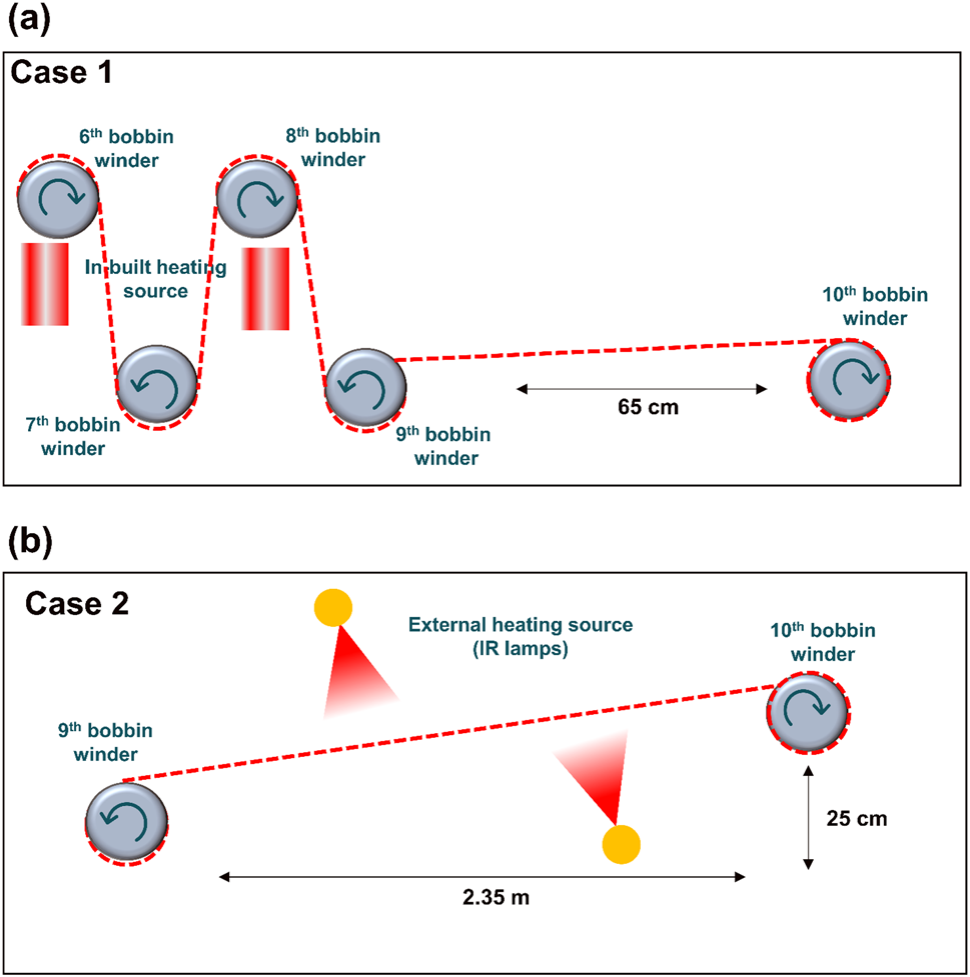
The positions of the tenth bobbin winder from the ninth bobbin winder (**a**) Case 1: initial distance of 65 cm away and (**b**) Case 2: optimized distance of 2.35 m away.

The strength, toughness, and shape of fabricated NCLFs in a continuous wet-spinning system highly depend on the position of the collector bobbin winder^24^. The optimal spinning speed of 30 cm/min used in the previous research was selected to begin the experiment to determine the initial spinning speed compatible with the integrated wet-spinning system^21^. The robustness of the RPM set for bobbin winders configured to match the spinning speed and the stability of the integrated wet-spinning system were assessed based on the system’s capacity to produce continuous NCLFs without breakage for an hour. Since the 30 cm/min spinning speed for the integrated wet-spinning system is relatively low, the NCLFs were air-dried before being wound onto the collector bobbin winder. The position of the collector bobbin winder is as shown in Case 1 of Fig. 2a. As a result, neither built-in nor external heaters were required for NCLF drying. The observed drying phenomenon can be attributed to the increased number of bobbin winders, which prolonged the time NCLFs took to reach the collector bobbin winder compared to the pilot wet-spinning system, which only had three bobbin winders. The spinning speed was increased to 100 cm/min to reduce the time required for the NCLFs to reach the collector bobbin winder and to prevent NCLFs from air-drying. The bobbin winders’ speed was again discreetly modified to match the spinning speed of 100 cm/min. The third built-in heater was set to 50 °C for the drying of NCLFs. The collector bobbin winder’s position remained constant, as in Case 1. The spinning speed was further increased to 180 cm/min to optimize the position of the collector bobbin winder to address NCLF drying and morphology with Case 2 (Fig. 2b). For NCLF drying, two out of three external IR lamps were used while the built-in heaters were turned off.

The NCLF output speed is related to the angular velocity of the bobbin:

1 $$ v = 2\pi r*N $$

where $v$ is the NCLF output speed (m/min), $N$ is the RPM of the bobbin winder and *r* is the radius of the collector bobbin winder (m). For the selected spinning speed of 180 cm/min, the NCLF output speed is 1.69 m/min, which is low for continuous production. Further, to increase the productivity of NCLF, three different spinning speeds: 250, 320, and 510 cm/min, were studied. Notably, a certain degree of stretching is inevitable while configuring the bobbin winder speeds to match the applied spinning speed to ensure the successful production of NCLF without breakage. Table 1 represents the parameter configuration of the integrated wet-spinning system.

**Table 1:** Parameter configuration of the integrated wet-spinning system for continuous NCLF fabrication.

Spinning speed (cm/min)	Position configuration	Built-in heater temp. (°C)	External IR lamp used (250 W)	Stretching ratio (%)
30	Case 1	–	–	–
100	Case 1	50	–	–
180	Case 2	–	2	5.5
250	Case 2	–	2	4.6
320	Case 2	–	2	5.5
510	Case 2	––	3	4.9

### Characterization

The morphology of fabricated NCLFs in the integrated wet-spinning system was characterized using a scanning electron microscope (SEM, JSM-6400 F, JEOL, Japan). An ion sputter coater (K575x, EMITECH, France) was utilized to deposit a thin platinum layer on the specimens before imaging.

Mechanical testing of the fabricated NCLFs was performed under the ASTM D-882–97 standard using a universal testing machine (Unitest, TESTONE, South Korea). Ten specimens were prepared and tested for each condition to analyze mechanical properties. A 5 kgf load cell was utilized for the test. The specimen gauge length was kept at 10 mm, and a constant strain rate of 0.15 mm/min was selected for the test. The relative humidity and temperature were maintained at 15% and 25 °C throughout the test. The area of NCLFs was measured from the fracture surface of SEM cross-section images of the specimen using software (Davo lite ver. 1.0).

## Results and discussion

### Optimization of spinning speed to enhance NCLF productivity

**Figure 3:**
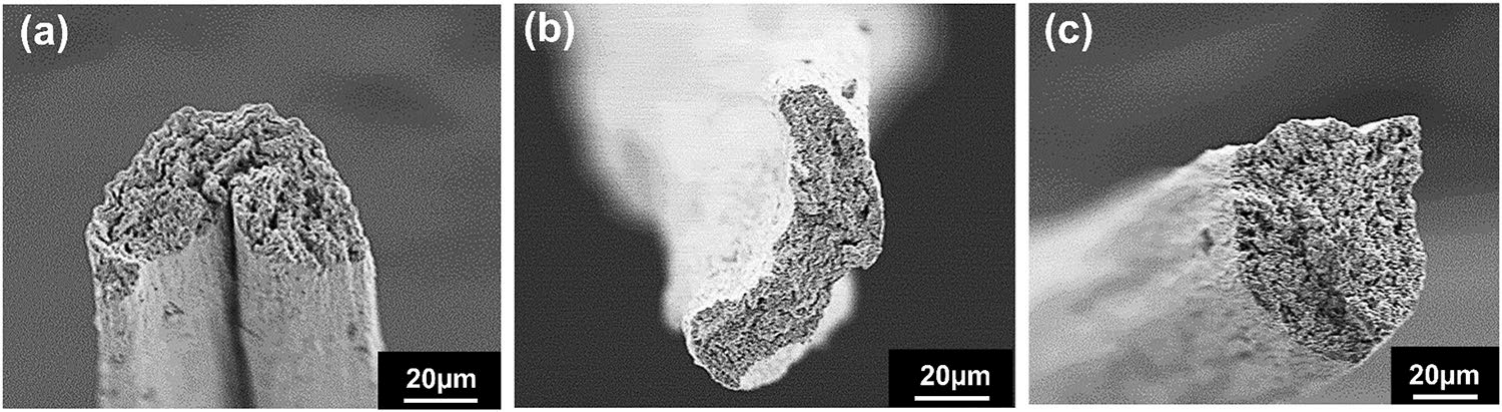
Cross-section SEM image of NCLFs fabricated with the spinning speed of (**a**) 30 cm/min, (**b**) 100 cm/min, and (**c**) 180 cm/min.

The 30 cm/min spinning speed was selected for experimental Case 1^21^. The RPM of each bobbin winder was individually configured to ensure continuous movement of NCLFs through the bobbin winders without breakage. Mechanical testing and morphological analysis were performed on the fabricated NCLFs collected from the collector bobbin winder to determine the quality of the NCLFs. The results indicated that the strength and toughness of the NCLFs were substantially compromised compared to the NCLFs fabricated in the pilot wet-spinning system. The NCLFs’ cross-section SEM images also revealed an irregular shape (Fig. 3a). The relatively low mechanical characteristics of these NCLFs can be related to weak hydrogen bonding between the NCLFs produced by sluggish air-drying while constantly moving through the bobbin winders. The drying time and temperature are considered important parameters in determining the strength of fabricated NCLFs. The abnormal pre-drying conditions may contribute to the formation of microbubbles inside NCLFs, resulting in defective NCLFs^26^. As shown in Fig. 3b, the NCLFs fabricated with a 100 cm/min spinning speed showed comparable morphology with NCLFs fabricated at 30 cm/min, which can be attributed to poor drying conditions.

Thus, the spinning speed was further increased to 180 cm/min to optimize the position of the collector bobbin winder with the Case 2 configuration. Since the spinning speed of 180 cm/min is relatively high compared to the 30 and 100 cm/min, it gives more scope to explore the effect of NCLF drying by external IR lamps on the mechanical properties of NCLFs. Figure 3c represents the cross-sectional SEM images of NCLFs fabricated at 180 cm/min. The cross-section image of NCLF fabricated at 180 cm/min reveals a preferred circular-shaped NCLF compared to previous trials. The abnormal morphologies of the NCLFs from 30 and 100 cm/min can be ascribed to the poor drying conditions and inappropriate positioning of the collector bobbin winder.

**Figure 4:**
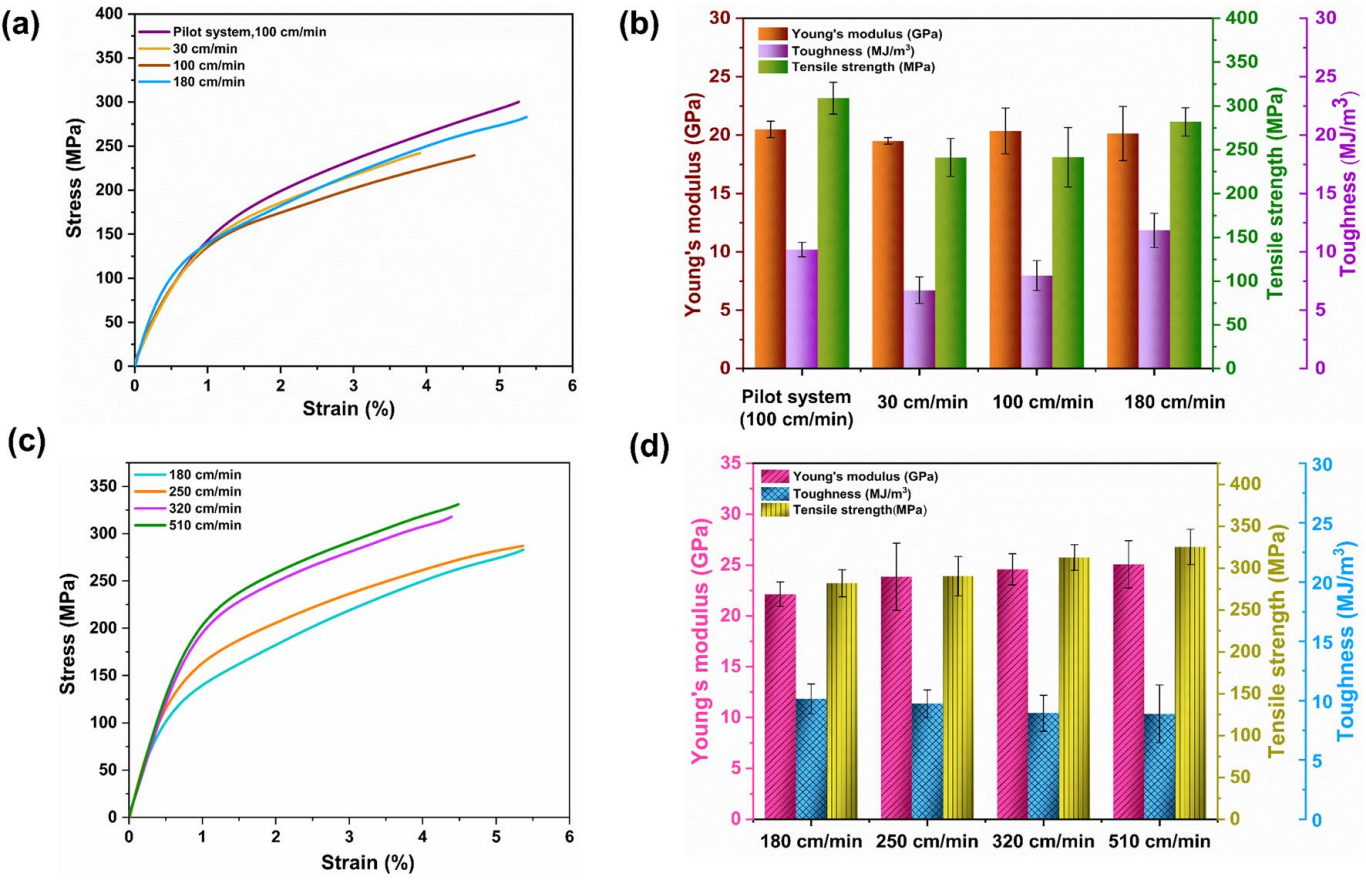
(**a**) Stress–strain curves of NCLFs fabricated at different spinning speeds in comparison to NCLF fabricated using the pilot system at 100 cm/min, (**b**) bar graphs summarizing the mechanical properties of fabricated NCLFs from each condition, (**c**) Stress–strain curve of NCLFs fabricated at different spinning speeds in the integrated wet-spinning system, and (**d**) bar graph summarizing the mechanical properties of fabricated NCLFs from each condition.

Figure 4a,b represents the stress–strain curves and mechanical properties of NCLFs with different spinning speeds. The NCLFs fabricated at a spinning speed of 180 cm/min exhibited a tensile strength of 281.8 MPa and toughness of 11.8 MJ/m^3^ without compromising strain at break (5.2%). From all tested spinning speeds, it is observed from the morphology and mechanical property analysis that 180 cm/min is the initial spinning speed compatible with the integrated wet-spinning system. It is also observed that spinning speeds below 180 cm/min might alter the quality of the NCLFs as it can impede the NCLF drying conditions. Also, the position of the collector winder, as shown in Fig. 2b, was ideal as it allowed NCLF drying using external IR lamps, resulting in high-quality NCLFs^24^. It can be attributed to the perfect NCLF drying without interruptions between the ninth and collector bobbin winders by offering a single horizontal plane. Contrarily, NCLFs constantly move through the bobbin winder when being dried using the built-in heaters, significantly reducing their mechanical strength as they are disturbed in their wet state^24^. Moreover, Young’s modulus, tensile strength, and toughness of the NCLFs fabricated at 180 cm/min in the integrated wet-spinning system are comparable to the NCLFs fabricated in the pilot system at 100 cm/min.

Increasing the spinning speed results in enhanced productivity of NCLF. Thus, the spinning speed of 250 cm/min was chosen, which is faster than 180 cm/min, and the bobbin winder speed was carefully tuned to match the spinning speed. The mechanical properties and morphology of NCLFs were analyzed to determine the quality of the NCLF produced by the selected spinning speed. The results indicated that the fabricated NCLFs show similar behavior to the NCLFs previously produced with the spinning speed of 180 cm/min, with a slight improvement in mechanical properties. The morphology analysis by SEM also revealed that the NCLFs also retained the preferred circular shape. Following these findings, 320 and 510 cm/min spinning speeds were attempted to enhance NCLF productivity. NCLFs were fabricated after setting the bobbin winder speed to match the chosen spinning speeds of 320 and 510 cm/min. Comparable to the results shown by NCLFs fabricated at spinning speeds of 180 and 250 cm/min, the mechanical properties and morphologies of the NCLFs fabricated at spinning speeds of 320 and 510 cm/min also exhibited excellent results. Thus, these spinning speeds are confirmed desirable for continuously producing high-strength NCLFs.

**Figure 5:**
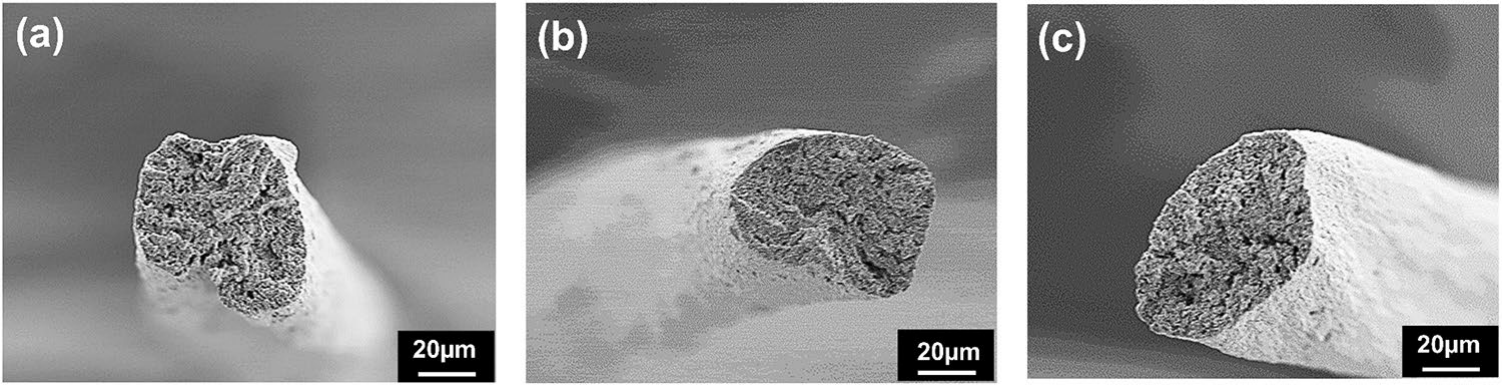
SEM cross-section images of NCLFs fabricated under spinning speeds (**a**) 250, (**b**) 320, and (**c**) 510 cm/min.

Figure 4c,d depicts the stress–strain curves and the mechanical properties of NCLFs fabricated at different spinning speeds. Table 2 summarizes the mechanical properties of NCLFs fabricated at different spinning speeds. The mechanical properties of NCLFs have significantly increased as the spinning speed increases. The progressive trend in the mechanical properties of NCLFs can be ascribed to the effect of shear stress generated inside the needle with increasing spinning speed, which promotes the reduction in NCLF area^10^. The Young’s modulus and tensile strength of NCLFs fabricated at 510 cm/min spinning speed were improved by 24% and 15%, respectively, compared to the NCLF fabricated at 180 cm/min. The strain at break and toughness of NCLFs are maintained in the expected range for all the attempted spinning speeds. The shapes of the NCLFs fabricated at spinning speeds of 250, 320, and 510 cm/min also reveal a preferred near-circular shape from the SEM cross-section images (Fig. 5).

**Table 2:** Mechanical properties of NCLFs fabricated under different spinning speeds.

Conditions	Young’s Modulus (GPa)	Yield strength (MPa)	Tensile Strength (MPa)	Strain at Break (%)	Toughness (MJ/m^3^)	Average area (µm^2^)
Pilot system (100 cm/min)	20.5 ± 0.7	162.4 ± 27.1	308.9 ± 18.1	4.8 ± 0.3	10.2 ± 0.6	696.3
30 cm/min	19.5 ± 0.3	147.5 ± 8.6	241.2 ± 21.7	4.0 ± 0.4	6.7 ± 1.1	537.3
100 cm/min	20.4 ± 2.0	138.9 ± 12.2	241.4 ± 33.9	4.7 ± 0.9	8.0 ± 1.3	479.0
180 cm/min	20.1 ± 1.2	153.4 ± 11.0	281.8 ± 16.1	5.2 ± 1.7	11.8 ± 1.5	687.7
250 cm/min	23.9 ± 3.3	184.7 ± 16.3	290.4 ± 23.5	5.4 ± 0.6	11.4 ± 1.4	652.6
320 cm/min	24.6 ± 1.5	193.2 ± 11.9	312.5 ± 15.4	4.9 ± 0.8	10.4 ± 1.8	633.5
510 cm/min	25.1 ± 2.3	217.0 ± 25.7	325.3 ± 21.1	4.5 ± 0.7	10.4 ± 2.8	612.9

For the experiments with different spinning ranging from 250 to 510 cm/min, only the external IR lamps were employed for NCLF drying. Additionally, the stability of the integrated wet-spinning system to continuously produce NCFLs with higher spinning speeds (320 and 510 cm/min) was also evaluated by its ability to maintain continuous production of NCLFs for an hour. The integrated wet-spinning system has proven stable with all the tested spinning speeds. With the increasing spinning speeds to 250, 320, and 510 cm/min, the NCLF output has dramatically improved to 2.39, 3.13, and 4.99 m/min, respectively. The productivity of NCLF has improved by five times with the integrated wet-spinning system compared to the pilot system. Figure S2 depicts the NCLF fabricated at the maximum applied spinning speed of 510 cm/min and collected on the collector roller. The NCLF production rate can further be improved by increasing the spinning speed and incorporating more heating sources for NCLF drying.

### Effect of electric field and mechanical stretching

**Figure 6:**
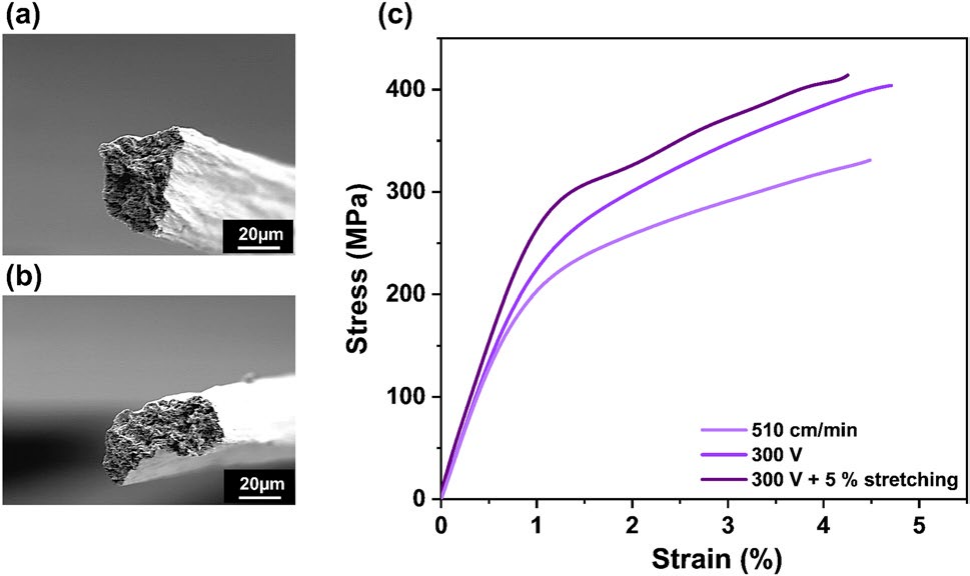
Cross-section SEM images of NCLFs fabricated under (**a**) AC electric voltage of 300 V, (**b**) with 300 V and 5% stretching (**c**) stress–strain curve of fabricated NCLFs from each condition.

The influence of external NCLF alignment techniques, such as AC electric field and mechanical stretching, was also investigated using the integrated wet-spinning system to explore its flexibility and adaptability. Following prior research, an AC electric field of 300 V (100 Hz) was introduced during the wet extrusion of the CNF suspension^21^. The initial spinning speed was maintained at 510 cm/min (4.9% stretching), and an additional 5% stretching was introduced between no. 3 and no. 9 bobbin winders in conjunction with NCLF fabrication using the 300 V. Figure 6a,b show the SEM cross-section images of NCLFs fabricated under 300 V and NCLFs fabricated by the combination of 300 V and 5% stretching, respectively. It is evident from Fig. 6b that, as NCLF alignment increases under AC electric voltage and stretching, the area of NCLF decreases; hence, the NCLF shape change from circular to oval^21^.

Figure 6c shows the stress–strain curves of the NCLFs fabricated by incorporating 300 V and 5% stretching, compared with the NCLF fabricated without AC field and stretching (510 cm/min). Table 3 summarizes the mechanical properties. A significant improvement in the mechanical properties is attributed to the area reduction of the NCLFs under the AC field^21^. The observed result can be attributed to the tight packing of individual CNFs in the NCLF and the formation of strong hydrogen bonding facilitated by appropriate drying conditions. The Young’s modulus, tensile strength, and toughness of NCLFs fabricated under 300 V and 5% stretching combination displayed superior properties among all the tested conditions.

**Table 3:** Mechanical properties of NCLFs fabricated under the spinning speed of 510 cm/min by incorporating an AC electric voltage of 300 V and 5% stretching.

Conditions	Young’s Modulus (GPa)	Yield strength (MPa)	Tensile strength (MPa)	Strain at break (%)	Toughness (MJ/m^3^)	Average area (µm^2^)
300 V	27.9 ± 0.0	229.2 ± 26.7	403.8 ± 20.1	4.6 ± 0.3	13.3 ± 0.5	512.6
300 V + 5% stretching	29.20 ± 0.8	270.0 ± 15.6	413.8 ± 15.0	4.2 ± 0.2	12.5 ± 1.1	491.2

## Conclusion

An integrated wet-spinning system was custom-designed to fabricate continuous high-strength NCLF on a laboratory scale. The developed wet-spinning system was tested by increasing the spinning speed and tuning key parameters, such as bobbin winder speed, collector bobbin winder position, and NCLF drying condition. Mechanical properties and morphology of the fabricated NCLFs were tested to assess the system performance. The spinning speed of the integrated wet-spinning system was increased to 180 cm/min by tuning other parameters, and it was further increased to 250, 310, and 510 cm/min to improve the NCLF productivity. The highest spinning speed of 510 cm/min resulted in the maximum NCLF production rate of 4.99 m/min, five times higher than the previous pilot system’s (0.92 m/min), with superior mechanical properties compared to CNF fabricated using other spinning systems. The synergetic effect of electric field and mechanical stretching on NCLF alignment was also studied to demonstrate the versatility of the integrated wet-spinning system. This research demonstrates the possibility of the integrated wet-spinning system for mass production of nanocellulose-based long filaments, with adaptability in being tailored for application-specific properties. The system can also be adapted for the large-scale production of multifunctional nanocellulose filaments with hybrid materials. Additionally, this integrated wet spinning system is designed to be flexible and adaptable, enabling the large-scale production of diverse filaments by facilitating pristine and hybrid suspensions.

### Supplementary Information

Supplementary Information.

## Data Availability

The datasets generated during and/or analyzed during the current study are available from the corresponding author upon reasonable request.
