# Supplementary material for: An integrated wet-spinning system for continuous fabrication of high-strength nanocellulose long filaments
Source: Sci Rep. 2023 Aug 12;13:13137. doi: 10.1038/s41598-023-40462-5 (PMC10423198; doi:10.1038/s41598-023-40462-5)
Supplement: Supplementary file 1 — Supplementary Information. [file 41598_2023_40462_MOESM1_ESM.docx]

**Supporting Information**

**An integrated wet-spinning system for continuous fabrication of high-strength nanocellulose long filaments**

Pooja S. Panicker^†^, Hyun Chan Kim^‡^, Jaehwan Kim^*†^

^†^Creative Research Center for Nanocellulose Future Composites, Inha University, Incheon 22212, Republic of Korea

^‡^ University of Michigan, Ann Arbor, MI 48109, USA

*Corresponding Author: Prof. Jaehwan Kim

Address: Creative Research Center for Nanocellulose Future Composites, Inha University, 100 Inha-ro, Michuhol-ku, Incheon 22212, Republic of Korea

Tel: +82-32-860-7326; Fax: +82-32-832-7325; Email: [jaehwan@inha.ac.kr](mailto:jaehwan@inha.ac.kr)


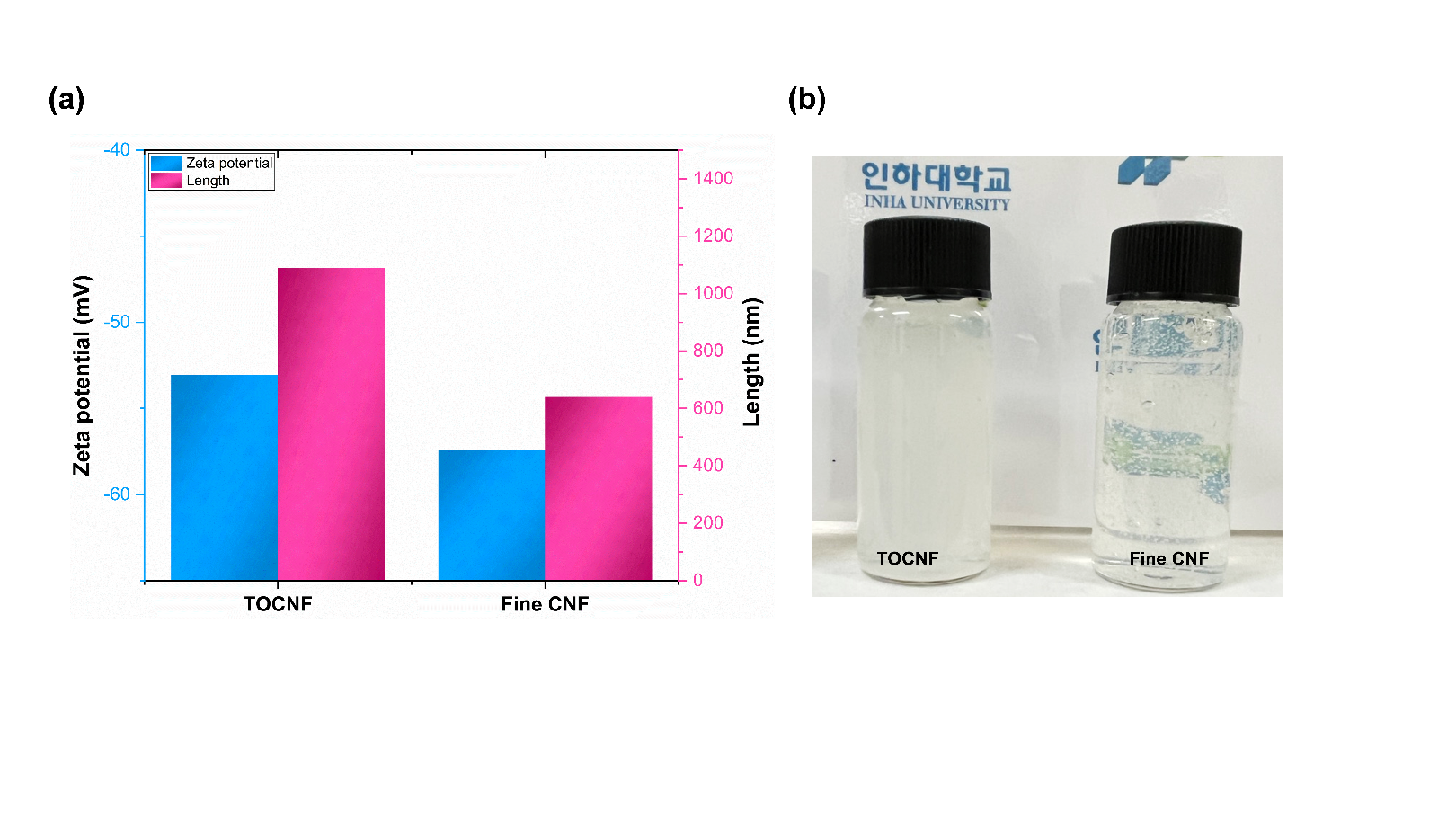


**Figure S1: (a)** Zeta potential and fiber length of TOCNF (tempo-oxidized CNF) and Fine CNF suspension and **(b)** photos of TOCNF and fine CNF

From **Figure S1**, it is evident that the zeta potential of fine CNF (-57.38) is higher than that of TOCNF (-53.05), and particles with a high zeta potential in a dispersion tend to have a more uniform distribution. The electrostatic repulsion or attraction between particles in a liquid media is reflected by the zeta potential. Electrostatic forces between particles with a high zeta potential are strong, resulting in greater repulsion. This repulsion keeps the particles from colliding or aggregating with one another. As a result, the particles are disseminated more uniformly throughout the liquid, resulting in a more equal distribution. The fiber length has also been reduced after ultracentrifuge with a narrow size distribution which directly influences the quality of fine CNF suspension ^1^.


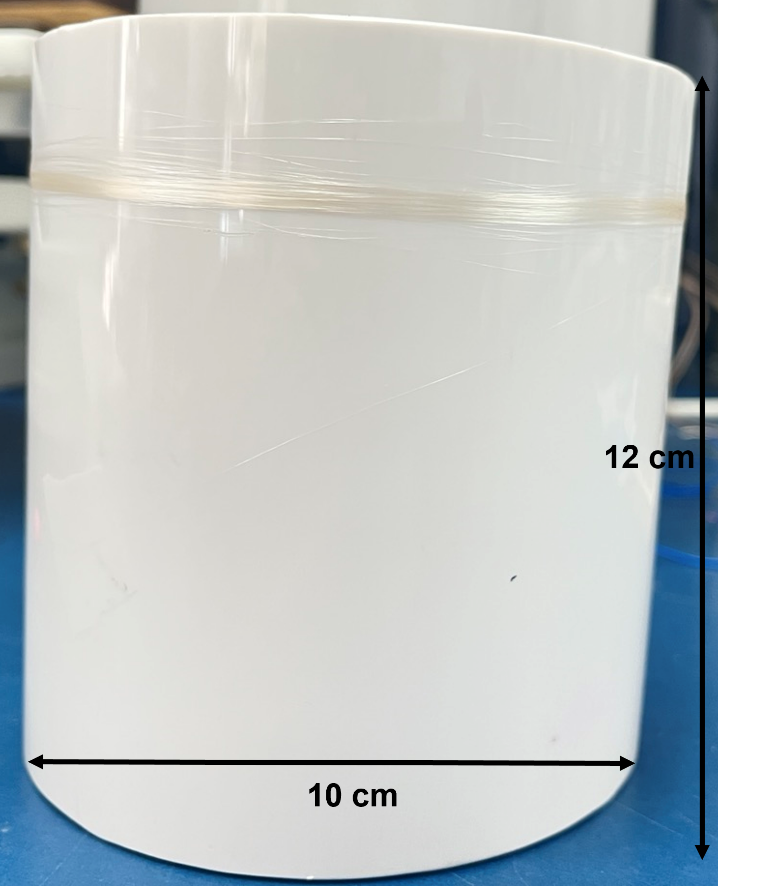


**Figure S2:** A photograph of the produced NCLF at 510 cm/min

**References**

1. Zhai, L., Kim, H. C., Kim, J. W. & Kim, J. Simple centrifugal fractionation to reduce the size distribution of cellulose nanofibers. *Sci. Rep.* **10**, 11744 (2020).
